# Diet Quality—The Greeks Had It Right!

**DOI:** 10.3390/nu8100636

**Published:** 2016-10-14

**Authors:** John J. B. Anderson, David C. Nieman

**Affiliations:** 1 Department of Nutrition, Schools of Public Health and Medicine, University of North Carolina at Chapel Hill, Chapel Hill, NC 27599, USA; jjb_anderson@unc.edu; 2 Human Performance Laboratory, Appalachian State University, North Carolina Research Campus, Kannapolis, NC 28081, USA

**Keywords:** polyphenols, Mediterranean diet, disease risk factors

## Abstract

The Mediterranean diet is upheld in the 2015–2020 Dietary Guidelines as an example of an eating pattern that promotes good health, a healthy body weight, and disease prevention throughout the lifespan. The Mediterranean eating pattern is based on a variety of unprocessed plant foods including fruits, vegetables, whole grains, legumes, nuts, and seeds that are high in polyphenols. The majority of polyphenols arrive in the colon where bacteria degrade them into smaller phenolics that can be translocated via the portal vein to the liver. In the liver, the phenolics undergo additional biotransformation prior to release into the circulation and transport to specific tissues where bioactive effects take place before removal in the urine. Recent epidemiologic studies using improved assessment techniques support that high versus low dietary polyphenol intake predicts reduced risk for neurodegenerative diseases, diabetes, cardiovascular disease, hypertension, obesity, and early death from all causes. Emerging science reveals that many of these health-related benefits can be traced to the biotransformed, gut-derived phenolics. In conclusion, the high consumption of unprocessed plant foods by inhabitants of countries bordering the Mediterranean Sea has been linked to multiple health and disease prevention benefits that are in large part due to a varied intake of polyphenols.

## 1. Introduction

In today’s modern way of living, as a society we have lost our bearings when it comes to healthy eating patterns. A high quality diet is based on a variety of foods, mostly from plant sources, and foods that are exposed to limited processing—the classical pattern of eating practiced by Greeks and other Mediterranean populations. A primary goal of the 2015–2020 Dietary Guidelines is that individuals throughout all stages of the lifespan have eating patterns such as the healthy Mediterranean diet that promote good health, a healthy body weight, and disease prevention [1]. 

Excesses of any component of the diet were typically avoided and consequently over-consumption was rare. Sugar, sweet desserts, and salt were rarely consumed, and animal products except for seafood and yogurt and cheese were less frequently consumed. In addition, olives and olive oil, nuts, and seeds were parts of daily intakes, as wine and spices often were. Balanced eating patterns of the macronutrients greatly reduced the development of overweight or obesity and metabolic diseases resulting from excessive caloric intakes. Intake percentages of fats and carbohydrates were fairly high, but total calories were not higher than maintenance requirements.

Currently, many in the Western world are fighting the battle of the bulge—body weight maintenance has gone awry. Balanced macronutrient intakes remain far too uncommon and processed foods, often laden with sugar or salt, or even possibly both, have become regular components of our diets. Average body weights of adults, and increasingly of children, have risen as a result of excessive caloric consumption combined with prolonged periods of physical inactivity [2]. In some nations, this enormous problem has had a slowing effect on their population longevity rates because of increasing mortality rates related to metabolic diseases. 

What can be done? A huge educational challenge is before us if we are going to be able to modify dietary practices of entire populations. A new emphasis on the meaning of diet quality must be instilled. Both adults and children need to buy into their own lifetime health as part of their everyday lives and our institutions need to be supportive in all ways. The issue of diet quality needs the investment of national governments and food industry members. As emphasized in the 2015–2020 Dietary Guidelines for Americans, all segments of society—individuals, families, communities, businesses and industries, organizations, governments, and others—can and should become involved [1]. In addition, the World Health Organization urges that what is needed is a “whole-of-government approach” [2]. A primary objective is to establish and maintain settings (e.g., homes, schools, worksites, restaurants, stores) that support and encourage food and beverage choices that help individuals make shifts to meet the key recommendations for healthy eating patterns. Accomplishment of the steps of instituting and sustaining healthy nutritional programs has occurred in Finland after recognition that the type 2 diabetes rates were far too excessive for an economically advanced nation [3].

Practical steps can be taken via emerging public health messages that utilize modern electronic communication techniques that focus on diet quality and other healthy lifestyle behaviors, including participation in regular physical activities and reducing smoking and alcohol consumption. First, the components of a quality diet need to be established.

## 2. Food Components of a Quality Diet and Health Benefits

The classical Mediterranean diet serves as a good example for eating healthy, using the basic food components, and the Mediterranean pattern can be adopted practically everywhere [4,5,6]. Mediterranean food guides are available [1] and variations—though possibly known by other names—can be used to make good food selections (Table 1), but the preparation of specific dishes may require instruction. Food markets should carry the fruits and vegetables that are in season; costs of these plant foods may be a question for those with stringent budgetary limits. Fish and other seafood may also challenge consumers with regard to cost. Nonetheless, most studies show that greater adherence to the Mediterranean dietary pattern can be achieved without incurring significantly greater costs, and that practically all households can adapt to this pattern of healthy eating if adequately motivated [6,7].

Several chronic diseases or conditions may benefit from the regular consumption of the Mediterranean dietary pattern of eating including cardiovascular disease (CVD), diabetes, and several types of cancers. The PREDIMED trial (Prevención con Dieta Mediterránea) showed that risk for CVD, diabetes, breast cancer, and atrial fibrillation was reduced in a group of individuals at high cardiovascular risk when consuming a Mediterranean diet supplemented with extra-virgin olive oil or nuts [8,9,10,11,12,13]. Additionally, both Mediterranean diets in the PREDIMED primary prevention trial improved multiple CVD risk factors including blood pressure, insulin sensitivity, lipid and lipoprotein profiles, inflammation, oxidative stress, and carotid atherosclerosis (see review, [8]). PREDIMED investigators concluded that these data provide strong evidence that a vegetable-based Mediterranean diet rich in unsaturated fat and polyphenols is a sustainable and ideal dietary pattern for CVD prevention [8]. The specific dietary components that confer these benefits have not yet been established, but presumably unsaturated fatty acids from seafood and olives, and dietary fiber, antioxidants, and polyphenols from the variety of fresh fruits and vegetables help prevent or delay adverse chronic disease conditions [5,8,10]. The healthfulness of the Mediterranean dietary pattern should be based more on overall food group intakes than on meeting specified nutrient standards, as emphasized in the 2015–2020 Dietary Guidelines for Americans [1]. 

Much research remains before specific dietary components can be singled out as disease-protective, but numerous plant molecules or phytochemicals are under investigation as critical components of disease-preventive diets. Distinctions between good fats and less healthy fats, and between good carbohydrates and harmful carbohydrates still need to be established. Nevertheless, high quality diets contain low levels of added sugars and readily digestible starches while at the same time providing adequate amounts of mono- and poly-unsaturated fats. For example, the Mediterranean patterns of eating are not low in total fat, only in saturated fat and trans-fats. In addition, these diets are high in polyphenols and unprocessed carbohydrates with dietary fiber, and low in added sugars, including high-fructose corn syrup. The high-plant food pattern of eating contributes to lower mortality rates, risk reductions of multiple chronic diseases, and control of metabolic disease risk factors [5,8,14]. Despite mounting evidence of these health benefits, U.S. adults average only two servings per day of fruits and vegetables, well below recommended levels [15].

## 3. Plant Foods for the Promotion of Health and Prevention of Chronic Diseases

What is so beneficial about fruits, vegetables, whole grains, and other plant items? Cereal grains tend to be rich in fiber and fruits and vegetables are primary sources of polyphenols. Plant foods in general are good sources of a number of nutrients, including complex carbohydrates, healthy types of fatty acids, and vitamins and minerals. These foods provide in their basic matrix, when raw or after limited processing, plant chemicals that aid in the support of body functions conducive to good health. Phytochemicals are non-nutritional bioactive substances found in vegetables, fruits, grains, nuts, and seeds. A large number of phytochemicals have been identified, but for many of them the potential beneficial effects are still unknown. Phytochemicals can be classified according to their chemical structure, and include carotenoids, polyphenols, nitrogen-containing compounds, and organosulfur compounds [16]. Most current studies have focused on the health benefits of polyphenols, in part due to the excellent databases that are now available through the USDA [17] and Phenol Explorer [18]. 

The long-held hypothesis that diets high in grain fibers help prevent colon cancer may no longer be supported by research studies, but clearly interactions of the different fiber types with gut bacteria do impact on the overall health of gastrointestinal (GI) tissue and chronic diseases. Now, a more general hypothesis holds that the phytochemicals plus adequate amounts of nutrients support gut health and more optimal function of other organs such as the liver and heart, but also the brain [19]. The gut microbiome is clearly affected by plant foods [18,19], but so far little is known about the specific bacterial species that are responsible for the beneficial actions of the different fiber types and the diverse polyphenols [20]. What we do know is that the gastrointestinal microbiome is flooded with energy in the form of undigested and partially digested food components each day. The more diverse the diet, the more diverse the microbiome, a critical attribute related to health and disease prevention, according to many recent studies [21,22]. 

**Figure 1:**
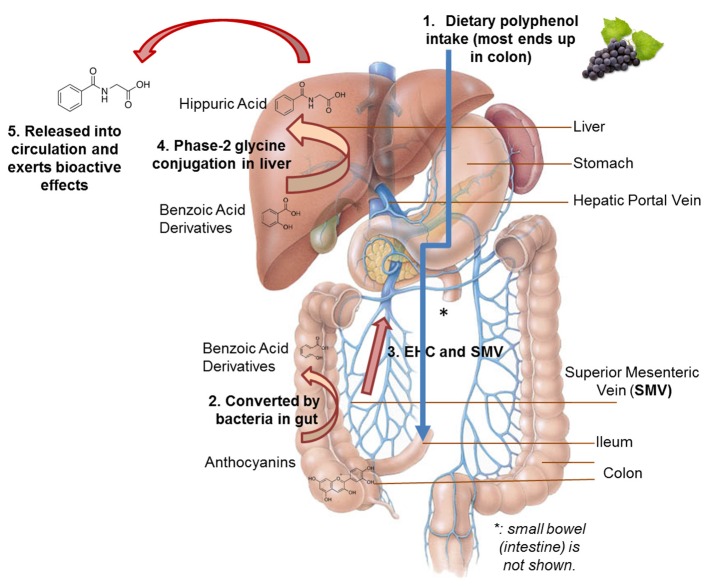
Polyphenol digestion and modification using purple grape anthocyanins as the model, with transformation to benzoic acid derivatives and hippuric acid [29,30]. Other polyphenols from diverse fruits and vegetables undergo similar metabolic modifications, resulting in many types of gut-derived phenolics.

Polyphenols have many beneficial roles, including anti-oxidation, anti-inflammation, and cell signaling [23,24,25]. In addition, they may contribute to weight management in adults [26]. High polyphenol diet intake, as assessed by recall or other methods, and elevated concentrations of total urinary polyphenols, a proxy measure of dietary intake, are predictive of reduced overall mortality rates in long-term epidemiological studies [27,28]. A large proportion of ingested plant polyphenols reach the colon and many metabolites generated by colonic bacterial enzymatic activity exert beneficial effects to colon endothelial cells before being absorbed into the systemic venous circulation from which they may exert additional bioactive effects prior to excretion in the urine [29,30,31]. The colonic bacterial transformation of food polyphenols varies widely depending on the unique gut microbiota composition of the individual as influenced by genotype and epigenetic variants, diet, lifestyle, and other factors [32]. Microbial degradation transforms the extremely diverse population of dietary polyphenols into a smaller number of metabolites, and these include simple phenols and derivatives of benzoic acid, phenylacetic acid, mandelic acid, phenylpropionic acid, and cinnamic acid [21,29,30,31,32,33,34]. Benzoic acid, for example, is a gut-derived end-product of polyphenolic degradation by bacteria that can be absorbed into the portal vein and routed to the liver. Liver cells then make hippuric acid by the addition of a small component to benzoic acid [35] (Figure 1). Hippuric acid exerts a variety of bioactive effects, and may act on various progenitor cells in the bone marrow that circulate in the blood to tissues throughout the body and act as a signal for new cell and tissue generation, particularly turning on bone cell lines and turning off fat cell lines [35,36,37,38]. Most hippuric acid produced is excreted in the urine and this excretory product serves as a marker of a good diet containing plentiful amounts of fruits and vegetables [38]. Phytochemicals in many fruits generate benzoic acid via gut bacterial activities [29,30,31,34,35].

Much remains to be learned about the potential beneficial effects of polyphenols from fruits, vegetables, and other plant foods in promoting health and preventing chronic diseases. Recent epidemiologic studies using improved assessment techniques support that high versus low dietary polyphenol intake predicts reduced risk for neurodegenerative diseases, diabetes, cardiovascular disease, hypertension, obesity, and early death from all causes [23,24,25,26,27,28,39,40,41,42]. A higher intake of flavonoids has been linked in the Nurses' Health Study to increased odds of healthy aging [43].

Recommendations for total dietary polyphenol intake and specific types of polyphenols and flavonoids have not yet been established but should be forthcoming in the near future as improvements in assessment methods continue [17,18,44,45]. Mean intake of flavonoids (the most abundant type of polyphenol in the diet) among US adults is 251 mg/day, with tea flavan-3-ols accounting for 81% of intake [44]. The high consumption of fruits, vegetables, unrefined cereals, legumes, nuts and seeds, and olive oil by inhabitants of countries bordering the Mediterranean Sea has been linked to a varied and healthful intake of flavonoids [45].

## 4. The Health Benefits of Plant Foods: A Lesson from the Greeks

Plant foods need to be considered as the base of any healthy diet because they provide not only the essential nutrients, but also the critically important health-promoting plant molecules (polyphenols) and dietary fiber now being investigated in major research studies throughout the world. Diet quality stems directly from these dietary plant sources. Exclusion of animal foods is not recommended. Certainly seafood and low-fat dairy products support health when consumed in moderate amounts on a regular basis, but red meat intakes should be reduced substantially to only a few servings a week rather than consumed every day. Emphasis needs to be placed on the health benefits of plant foods as the major components of a healthy diet in Western nations today because of both the nutrients and other components contained in them.

## Figures and Tables

**Table 1:** Mediterranean food and fluid guide (pyramid) [4].

Foods and Fluids	Consumption Level
Meats and sweet desserts—1 to 2 days a week	Low
Yogurt, low-fat cheese, poultry, and eggs—5 to 7 days a week	Moderate
Fish and seafood—5 to 7 days a week	Moderate
Nuts and seeds—every day	Moderate
Fruits, vegetables, whole grains, and legumes—every day	High
Olives and olive oil—every day	High
Herbs, spices, and garlic	High
Wine—2 servings/day (M) or 1 per day (F)	Moderate
Water	High
